# Mathematical Theory of Seismic Activity and Its Specific Cases: Gutenberg–Richter Law, Omori Law, Roll-Off Effect, and Negative Binomial Distribution

**DOI:** 10.3390/e27020130

**Published:** 2025-01-26

**Authors:** Roumen Borisov, Nikolay K. Vitanov

**Affiliations:** Institute of Mechanics, Bulgarian Academy of Sciences, Academician Georgi Bonchev Street, Block 4, 1113 Sofia, Bulgaria; r.borisov@outlook.com

**Keywords:** seismic activity, earthquakes, energy-based model, Gutenberg–Richter law, Omori law, negative binomial distribution, roll-off effect

## Abstract

We discuss a model of seismic activity that is based on the concept of energy in a cluster of sources of seismic activity. We show that specific cases of the studied model lead to the Gutenberg–Richter relationship and the Omori law. These laws are valid for earthquakes that happen in a single cluster of sources of seismic activity. Further, we discuss the distribution of earthquakes for several clusters containing sources of seismic activity. This distribution contains, as a specific case, a version of the negative binomial distribution. We show that at least a part of the roll-off effect connected to the parameter *b* of the Gutenberg– Richter law occurs because one records earthquakes that happen in more than one cluster of sources of seismic activity.

## 1. Introduction

Earthquakes are complicated and interesting space-time events. One reason for the large research interest in earthquakes is that the destruction potential of an earthquake can be very large. Because of this, it is very important for us to have methods for forecasting the probability that an earthquake will occur. This can help us make decisions in the case of emergencies that are connected to an earthquake [1].

Another reason for the research interest in earthquakes is the hypothesis that earthquakes are specific members of the class of critical phenomena [2,3,4]. A reason for this hypothesis is that the frequency of earthquakes has a power law relationship to quantities which are characteristic of the size and energy of an earthquake [5].

A standard test for evaluating models is to compare the number of forecast events to the number of real events for the tested period of time. To perform this task, one has to know the relationship for the probability distribution of earthquakes. A discussion of several discrete distributions can be found in [6], where the Poisson distribution, the geometric distribution, the logarithmic distribution, and the negative binomial distribution are discussed. Of these distributions, the negative binomial distribution is the only one that has two parameters. Kagan [6] assumes the possibility that the second parameter in the negative binomial distribution could describe the clusterization of the process.

Several empirical relationships are well-known in seismology. The Gutenberg–Richter law [7] gives a relationship between the magnitude and the number of earthquakes in a region for an interval of time:

(1) $$\log_{10}N=a-bM.$$

where *M* is the magnitude of the earthquake and N is the number of earthquakes with a magnitude equal to or larger than *M*. The magnitude of an earthquake reflects its strength, and it is measured through the magnitude recorded by seismometers. The parameter *a* depends on the total number of events and describes the seismic productivity of the region under consideration. The parameter *b* gives information about the ratio between strong and weak earthquakes in the region. The magnitude *M* reflects the size of an earthquake, but it is different from the strength of the quake (the energy *E* freed by the quake) and its seismic moment $M_{0}$. Empirically, the relationships between the magnitude, energy, and seismic moment of an earthquake are

(2) $$\begin{array}{ccc} \log_{10}M_{0} & = & \alpha_{0}+\beta_{0}M,\\ \log_{10}E & = & \alpha +\beta M, \end{array}$$

where $\alpha_{0},\alpha ,\beta_{0},\beta$ are empirical constants and $\beta \approx \beta_{0}=1.5$ [8]. Then

(3) $$N\propto M_{0}^{-}B_{0},\;\;\;N\propto E^{-B},$$

where $B_{0}=2b/3$, and it is analogous to the parameter *b* from the Gutenberg–Richter law [5,8,9].

In all empirical catalogs of earthquakes, a noticeable decline can be observed in the value of *b* for earthquakes of small magnitudes. This is referred to as the “roll-off” effect of the value of *b*. In the logarithmic plot of the Gutenberg–Richter law, the roll-off effect is seen as an almost horizontal curve in the region of small magnitudes. This may be a result of the incompleteness of the data in the area of small magnitudes due to a decrease in the experimentally recorded amplitude in relation to the amplitude of the noise [10]. Some recent models of earthquake dynamics lead to the roll-off effect as a natural consequence of the model [11,12].

In addition, due to the finite size of the regions of seismic activity, the Gutenberg–Richter law should be constrained from above. In other words, a maximum amplitude $M_{max}$ should exist for the considered space-time interval [13,14]. The value of $M_{max}$ is an important characteristic, as it is necessary for the evaluation of the risk associated with large earthquakes in the region under consideration. Thus, various methodologies for the determination of $M_{max}$ are available [15,16,17]. In order to account for the above-mentioned phenomena, some authors modify the Gutenberg–Richter law [18].

Another famous law in seismology is the Omori law of aftershocks [19]. The Omori law states that after a strong earthquake, the frequency of aftershocks (the underground shocks that follow the main shock) decays with time, on average, according to the hyperbolic law [20,21]:

(4) $$N^{*}\left(t\right)=\frac{k}{c+t},k>0,c>0.$$

Nowadays, one uses the formula [22,23]

(5) $$N^{*}\left(t\right)=\frac{k}{{\left(c+t\right)}^{p}},k>0,c>0,$$

and *p* can be larger than 1 or smaller than 1.

In this article, we discuss a simple model of seismic activity. The model does not contain assumptions about the mechanics of seismic events, such as the consideration of fractures. Instead, we base the model on considerations connected to energy. Our goal is to show that specific cases of the model lead to several well-known relationships and distributions in seismology such as the Gutenberg–Richter relationship, the Omori law, and the negative binomial distribution.

The text below is organized as follows. In Section 2, we formulate a model based on several assumptions about the motion of energy in a volume containing a cluster of sources of seismic activity. In Section 3, we discuss several results for specific cases of the model. Section 4 is devoted to the discussion of the energy–frequency distributions that occur on the basis of the model. In Section 5, we discuss the magnitude–frequency distributions and the conditions for the occurrence of the Gutenberg–Richter relationship. Section 6 is devoted to a discussion of the relationship between the model and the Omori law. We show in Section 7 that the negative binomial distribution of earthquakes can be connected to the recording of earthquakes from several regions of seismic activity containing different clusters of sources of seismic activity. A short discussion of the results is presented in Section 8. Several concluding remarks are summarized in Section 9.

## 2. Mathematical Formulation of the Problem

There are many studies on earthquakes based on an assumption of clustering and on stochastic point processes [24,25,26,27,28,29,30,31]. We mention, especially, the models based on branching processes [32,33,34,35,36,37,38]. Such models can reproduce the Gutenberg–Richter relationship and the Omori law. We also note the negative binomial distribution, which was used to approximate the earthquake numbers in catalogs [36,37,38,39,40,41,42]. We show below that a simple model of seismic activity can lead to similar results as those obtained by the models mentioned above. We have already studied models of this kind and have applied them to various problems connected to the motion of substances in networks [43,44,45,46,47,48,49].

Let us consider some volume below the surface of the Earth. In this volume, we assume the existence of a cluster of sources of seismic activity. We note the difference between the source of seismic activity and the seismic source. An earthquake is a seismic source. As a source of seismic activity, we understand a volume which contains a potential seismic source. Thus, below, we understand the energy of the source of seismic activity to be internal energy stored in the corresponding volume and connected to the potential seismic source.

Our model is simple. It is based only on the internal energy connected to a source of seismic activity. We do not consider below the complicated mechanics connected to the seismic sources [50]. We assume that the source of seismic activity may arise from an inflow of energy into the considered cluster. The energy of a source of seismic activity may increase or decrease. At some point, the energy can be released, and we observe a seismic event (an earthquake).

In our model, we collect the sources of seismic activity into groups with respect to the internal energy of the source. Let $E_{i}$ be the maximum internal energy of the *i*-th group of sources. Then, the sources from the initial group of sources have internal energy smaller than $E_{0}$. We denote this group of sources as the 0-th group of sources. The sources of the next group have internal energy larger than $E_{0}$ and smaller than or equal to $E_{1}$. This is the first group of sources of seismic activity. We further define the second group of sources (sources with internal energy up to $E_{2}>E_{1}$), etc. The intervals between $E_{i}$ and $E_{i+1}$ can be chosen to be small enough. Thus, we can have numerous intervals.

Several processes connected to the internal energy of the sources of seismic events are taken into account in the model. A source of seismic activity may arise or vanish in each of the groups. The reason for this is energy, which arrives from (or moves to) the environment. The sources of seismic activity can move from one group of sources to another group of sources. We assume that this motion is only between the neighboring groups of sources (as we discuss short intervals of time). The sources can move from group *i* to group i+1. Such sources of seismic activity gain energy. Other sources can move from group *i* to group i−1. Such sources of seismic activity lose energy. Finally, in each of the groups, a source of seismic events can release its energy and produce an earthquake.

The processes connected to our model are described mathematically as follows:

1. Arising of new sources of seismic activity: In the considered cluster for unit time, we observe the birth of $\sigma_{i}\left(t\right)N_{i}\left(t\right)$ new sources of seismic activity, where $N_{i}\left(t\right)$ is the number of sources in the *i*-th group in the time *t* (the number of sources of internal energy between $E_{i-1}$ and $E_{i}$). $\sigma_{i}>0$ corresponds to arising of new sources. $\sigma_{i}<0$ corresponds to the vanishing of sources.
2. Changes in the internal energy of the existing sources of seismic activity: Part $f_{i}\left(t\right)N_{i}\left(t\right)$ of the sources of seismic activity from the *i*-th group of sources of seismic activity moves to the i+1-st group of sources of seismic activity. Such changes lead to sources of seismic activity with greater internal energy.
3. Changes in the internal energy of the existing sources of seismic activity: Part $g_{i}\left(t\right)N_{i}\left(t\right)$ of the sources of seismic activity from the *i*-th group of sources of seismic activity moves to the i−1-st group of sources of seismic activity. These changes lead to sources of seismic activity with smaller internal energy.
4. Release of energy of sources of seismic activity: Part $\gamma_{i}\left(t\right)N_{i}\left(t\right)$ of sources of seismic activity from the *i*-th group of sources of seismic events releases its energy and an earthquake occurs. The seismic energy of this earthquake is assumed to be proportional to the internal energy of the corresponding source. We note that in the presented theory, the earthquakes are seismic sources. The energy released by an earthquake can move to other sources of seismic activity from the cluster and may create new sources of seismic activity.

We assume that changes in the energy occur continuously over time. The corresponding model equations are

(6) $$\begin{array}{ccc} \frac{dN_{0}}{dt} & = & \sigma_{0}\left(t\right)N_{0}\left(t\right)-\left[f_{0}\left(t\right)+\gamma_{0}\left(t\right)\right]N_{0}\left(t\right)+g_{1}\left(t\right)N_{1}\left(t\right),\\ \frac{dN_{i}}{dt} & = & \sigma_{i}\left(t\right)N_{i}\left(t\right)+f_{i-1}\left(t\right)N_{i-1}\left(t\right)-\left[f_{i}\left(t\right)+\gamma_{i}\left(t\right)\right]N_{i}\left(t\right)+g_{i+1}\left(t\right)N_{i+1}\left(t\right)-g_{i}\left(t\right)N_{i}\left(t\right),i=1,\ldots . \end{array}$$

The model (6) is a complicated one and can be treated numerically. It has an important specific case that can be treated analytically. In this case, $g_{i}\left(t\right)$ is very small, and because of this, these terms can be neglected. In other words, we assume that the probability that a source of seismic activity loses internal energy for reasons other than an earthquake is very small in comparison to the probabilities of occurrence for the other processes described in the model. In this case, the corresponding model equations become

(7) $$\begin{array}{ccc} \frac{dN_{0}}{dt} & = & \sigma_{0}\left(t\right)N_{0}\left(t\right)-\left[f_{0}\left(t\right)+\gamma_{0}\left(t\right)\right]N_{0}\left(t\right),\\ \frac{dN_{i}}{dt} & = & \sigma_{i}\left(t\right)N_{i}\left(t\right)+f_{i-1}\left(t\right)N_{i-1}\left(t\right)-\left[f_{i}\left(t\right)+\gamma_{i}\left(t\right)\right]N_{i}\left(t\right),i=1,\ldots . \end{array}$$

The solution of the model system of Equation (7) is as follows:

(8) $$\begin{array}{ccc} N_{0}\left(t\right) & = & C_{0}\exp\left[\int dt(\sigma_{0}\left(t\right)-f_{0}\left(t\right)-\gamma_{0}\left(t\right))\right],\\ N_{i}\left(t\right) & = & \exp\left[-\int dt\;\;[f_{i}\left(t\right)-\sigma_{i}\left(t\right)+\gamma_{i}\left(t\right)]\right]\{C_{i}+\int dt\;\;f_{i-1}\left(t\right)N_{i-1}\left(t\right)\exp([\int dt\;\;[f_{i}\left(t\right)-\\ & & \sigma_{i}\left(t\right)+\gamma_{i}\left(t\right)]])\}, \end{array}$$

where $C_{0}$ and $C_{i}$ are constants of integration. By means of this model, we can discuss many situations in the studied cluster of sources of seismic activity.

We note that we can also write equations for the finite number of groups i=0,1,…,I. In this case, we have again (7), and the second equation of this system is for i=1,…,I−1. For i=I, we have an additional equation. In this equation, we have to account for the fact that there is no next group, and because of this, $f_{I}=0$. The additional equation becomes

(9) $$\frac{dN_{I}}{dt}=f_{I-1}\left(t\right)N_{I-1}\left(t\right)+\left[\sigma_{I}\left(t\right)-\gamma_{I}\left(t\right)\right]N_{I}\left(t\right)-g_{I}\left(t\right)N_{I}\left(t\right).$$

As we assume that $g_{I}\left(t\right)$ has small values, (9) becomes

(10) $$\frac{dN_{I}}{dt}=f_{I-1}\left(t\right)N_{I-1}\left(t\right)+\left[\sigma_{I}\left(t\right)-\gamma_{I}\left(t\right)\right]N_{I}\left(t\right).$$

Next, we show that specific cases of the above model lead to the Gutenberg–Richter law and the Omori law, which are well known in seismology.

## 3. Several Specific Cases of the Model

First, we consider the specific case where the model coefficients are time-independent. In this case, the model system (7) becomes

(11) $$\begin{array}{ccc} \frac{dN_{0}}{dt} & = & \sigma_{0}N_{0}\left(t\right)-\left[f_{0}+\gamma_{0}\right]N_{0}\left(t\right),\\ \frac{dN_{i}}{dt} & = & \sigma_{i}N_{i}\left(t\right)+f_{i-1}N_{i-1}\left(t\right)-\left[f_{i}+\gamma_{i}\right]N_{i}\left(t\right),\;i=1,\ldots . \end{array}$$

The solution is a specific case of the solution to the case of time-dependent coefficients

(12) $$\begin{array}{ccc} N_{0}\left(t\right) & = & C_{0}\exp\left[\left(\sigma_{0}-f_{0}-\gamma_{0}\right)t\right],\\ N_{i}\left(t\right) & = & \exp\left[(\sigma_{i}-f_{i}-\gamma_{i})t\right]\left\{C_{i}+f_{i-1}\int dt\;\;N_{i-1}\left(t\right)\exp([f_{i}+\gamma_{i}-\sigma_{i}]t\right])\}, \end{array}$$

where $C_{0}$ and $C_{i}$ are constants of integration.

We will consider an even simpler specific case. This is a stationary case with time-independent model coefficients. In this case, the number of sources of seismic activity in each interval of energy does not change despite the changes in internal energy of the sources of seismic activity. The model equations are

(13) $$\begin{array}{ccc} \sigma_{0}N_{0}-\left[f_{0}+\gamma_{0}\right]N_{0} & = & 0,\\ \sigma_{i}N_{i}+f_{i-1}N_{i-1}-\left[f_{i}+\gamma_{i}\right]N_{i} & = & 0,i=1,\ldots . \end{array}$$

We use this specific case in the discussion below in the text.

In the stationary case, we have

$$\sigma_{0}-f_{0}-\gamma_{0}=0,$$

and $N_{0}$ is a free parameter. For the case of an infinite number of groups

$$N_{i}=\frac{f_{i-1}}{f_{i}+\gamma_{i}-\sigma_{i}}N_{i-1},i=1,\ldots$$

$N_{i-1}$ and $N_{i}$ must be constants because of $\frac{dN_{i}}{dt}=0$. Then, we can set $f_{i-1}=\kappa_{i}\left[f_{i}+\gamma_{i}-\sigma_{i}\right]$, where $\kappa_{i}$ is a parameter. We note that $\kappa_{i}<1$ for the case $N_{i}<N_{i-1}.$ For $N_{i}$, we obtain

(14) $$N_{i}=N_{0}\prod_{j=1}^{i}\kappa_{j}=N_{0}\prod_{j=1}^{i}\frac{f_{j-1}}{f_{j}+\gamma_{j}-\sigma_{j}},i=1,\ldots .$$

For the case of a finite number of groups, we obtain

$$N_{i}=N_{0}\prod_{j=1}^{i}\kappa_{i}=N_{0}\prod_{j=1}^{i}\frac{f_{j-1}}{f_{j}+\gamma_{j}-\sigma_{j}},i=1,\ldots ,I-1$$

and

(15) $$N_{I}=-\frac{f_{I-1}}{\delta_{I}}N_{I-1}=-\frac{f_{I-1}}{\sigma_{I}-\gamma_{I}}N_{0}\prod_{j=1}^{I-1}\kappa_{j}=\frac{f_{I-1}}{\gamma_{I}-\sigma_{I}}N_{0}\prod_{j=1}^{I-1}\frac{f_{j-1}}{f_{j}+\gamma_{j}-\sigma_{j}}.$$

Next, we can calculate the number of earthquakes in our system for the considered case of stationary-state and time-independent coefficients. For the case when the number of groups is infinite, the number of events by group *i* is

(16) $$N_{i}=\gamma_{i}N_{i}=\gamma_{i}N_{0}\prod_{j=1}^{i}\kappa_{j}=\gamma_{i}N_{0}\prod_{j=1}^{i}\frac{f_{j-1}}{f_{j}+\gamma_{j}-\sigma_{j}},i=1,\ldots .$$

For the case of a finite number of groups, we have again (16), but in this case, *i* changes from 1 to I−1. For i=I, we can use (16), keeping in mind that $f_{I}=0$.

We note that for the case $\kappa_{i}=\kappa$, (16) reduces to

(17) $$N_{i}=\gamma_{i}N_{i}=\gamma_{i}N_{0}\kappa^{i},i=1,\ldots .$$

In this case, $\kappa =\frac{f_{j-1}}{f_{j}+\gamma_{j}-\sigma_{j}}$.

Let us consider another specific case. This specific case would be of use for us in Section 7 when we discuss the relationships of the number of earthquakes arising from several regions. In this specific case, $\sigma_{i}=0$, i=1,… for the case of an infinite number of groups. This means that energy arrives at the system only through the smallest sources of seismic activity, which are in group 0. The model Equation (11) become

(18) $$\begin{array}{ccc} \frac{dN_{0}}{dt} & = & \sigma_{0}N_{0}\left(t\right)-\left[f_{0}+\gamma_{0}\right]N_{0}\left(t\right).\\ \frac{dN_{i}}{dt} & = & f_{i-1}N_{i-1}-\left[f_{i}+\gamma_{i}\right]N_{i}\left(t\right),i=1,\ldots . \end{array}$$

Let us consider the specific case when all sources of seismic activity of a group are active, and they either supply energy to the next group or release energy by an earthquake. Then $f_{i}+\gamma_{i}=1$ and we can write $f_{i}=q_{i}$ and $\gamma_{i}=1-q_{i}$. Then $\kappa_{i}=q_{i}$. We obtain from the model equations for the case of an infinite number of groups

(19) $$\begin{array}{ccc} \frac{dN_{0}}{dt} & = & \sigma_{0}N_{0}\left(t\right)-N_{0}\left(t\right).\\ \frac{dN_{i}}{dt} & = & q_{i-1}N_{i-1}-N_{i}\left(t\right),i=1,\ldots , \end{array}$$

For the stationary case, $N_{0}$ is a free parameter and $\sigma_{0}=1$. Further, we obtain for this case

(20) $$N_{i}=q_{i-1}N_{i-1}=N_{0}\prod_{j=0}^{i-1}q_{j}$$

For the case when all $q_{i}=q$, this reduces to

(21) $$N_{i}=N_{0}q^{i}.$$

The number of earthquakes in the case of different $q_{i}$ is

(22) $$N_{i}=\gamma_{i}N_{i}=\left(1-q_{i}\right)N_{0}\prod_{j=0}^{i-1}q_{j}.$$

For the case of all $q_{i}=q$, this reduces to

(23) $$N_{i}=\gamma_{i}N_{i}=\left(1-q\right)q^{i}N_{0}.$$

Let us now obtain the corresponding relationships for the case of a finite number of groups. In this case, the model equations are

(24) $$\begin{array}{ccc} \frac{dN_{0}}{dt} & = & \sigma_{0}N_{0}\left(t\right)-N_{0}\left(t\right),\\ \frac{dN_{i}}{dt} & = & q_{i-1}N_{i-1}-N_{i}\left(t\right),i=1,\ldots ,I-1,\\ \frac{dN_{I}}{dt} & = & q_{I-1}N_{I-1}-\left(1-q_{I}\right)N_{I}\left(t\right). \end{array}$$

Then, for the stationary case, we have again $N_{0}$ as a free parameter and (21) till i=I−1. For i=I, one obtains

(25) $$N_{I}=\frac{q_{I-1}}{1-q_{I}}N_{0}\prod_{j=0}^{I-2}q_{j}.$$

In the case $q_{i}=q$, this leads to

(26) $$N_{I}=\frac{q^{I}}{1-q}N_{0}.$$

The total number of earthquakes from category *I* in the case of a finite number of groups is

(27) $$N_{I}=\left(1-q_{I}\right)\frac{q_{I-1}}{1-q_{I}}N_{0}\prod_{j=0}^{I-2}q_{j}.$$

In the case $q_{i}=q$, this leads to

(28) $$N_{I}=q^{I}N_{0}.$$

The last results will be used in Section 7, where we will discuss earthquakes from more than one area and the connection of this situation to the negative binomial distribution.

## 4. Energy-Frequency Distribution

Let us return to the case of an infinite number of groups. We start from (14). We assume that the quantities connected to the exchange of energy depend on the internal energy, which is characteristic of the corresponding group. Let us assume the following relationships:

(29) $$\begin{array}{c} f_{i}=f_{i}^{*}E_{i}^{-s};\;\;\;\gamma_{i}=\gamma_{i}^{*}E_{i}^{-s};\;\;\;\sigma_{i}=\sigma_{i}^{*}E_{i}^{-s}. \end{array}$$

Above, $f_{i}^{*}$, $\gamma_{j}^{*}$, and $\sigma_{j}^{*}$ are constant parameters. *s* is also a parameter (we assume that this parameter has positive value). From (14), we obtain

(30) $$N_{i}=N_{0}\prod_{j=1}^{i}\frac{f_{j-1}^{*}}{f_{j}^{*}+\gamma_{j}^{*}-\sigma_{j}^{*}}{\left(\frac{E_{i-1}}{E_{i}}\right)}^{-s}$$

Let us denote

$$\chi_{i}\left(E_{i},E_{i-1}\right)=\prod_{j=1}^{i}\frac{f_{j-1}^{*}}{f_{j}^{*}+\gamma_{j}^{*}-\sigma_{j}^{*}}{\left(\frac{E_{i-1}}{E_{i}}\right)}^{-s}.$$

Then, for the number of earthquakes of category *i*, we obtain

(31) $$N_{i}=\gamma_{i}N_{i}=\gamma_{i}^{*}N_{0}\chi_{i}\left(E_{i},E_{i-1}\right)E_{i}^{-s}.$$

Note that if $\chi_{i}\left(E_{i},E_{i-1}\right)$ does not depend much on *i* and it is close to a constant, then $N_{i}\propto E_{i}^{-s}$.

We can choose different relationships for the relation between $E_{i}$ and $E_{i-1}$. For example, we can assume

$$E_{i}=\epsilon E_{i-1},\;\;\epsilon >1.$$

Then we have $\chi_{i}\left(E_{i},E_{i-1}\right)=\epsilon^{s}\prod_{j=1}^{i}\frac{f_{j-1}^{*}}{f_{j}^{*}+\gamma_{j}^{*}-\sigma_{j}^{*}}=\epsilon^{s}\chi_{i}^{*}$.

Thus, from (30), we obtain

$$N_{i}=N_{0}\chi_{i}^{*}\epsilon^{s}.$$

For the total number of earthquakes of category *i*, we obtain

(32) $$N_{i}=\gamma_{i}N_{i}=N_{0}\epsilon^{s}\chi_{i}^{*}\gamma_{i}^{*}E_{i}^{-s}.$$

We note that we can consider another relationship between $E_{i}$ and $E_{i-1}$. For example, we can assume $E_{i}=E_{i-1}+\Delta$ where Δ is some constant.

Then $\chi_{i}\left(E_{i},E_{i-1}\right)=\chi_{i}\left(E_{i}\right)=\epsilon^{s}\prod_{j=1}^{i}\frac{f_{j-1}^{*}}{f_{j}^{*}+\gamma_{j}^{*}-\sigma_{j}^{*}}{\left(1-\frac{\Delta }{E_{i}}\right)}^{-s}$ and

(33) $$N_{i}=\gamma_{i}N_{i}=N_{0}\chi_{i}\left(E_{i}\right)\gamma_{i}^{*}E_{i}^{-s}.$$

For small $\frac{\Delta }{E_{i}}$ from (33), we have again that $N_{i}\propto E_{i}^{-s}$.

## 5. Magnitude–Frequency Distribution. Gutenberg–Richter Law

The magnitude of the earthquake is proportional to the released energy. Thus, as magnitude $M>M_{i}$, we understand an earthquake with energy $E>E_{i}$.

Let us discuss the conditions for the occurrence of the Gutenberg–Richter law in our model. We begin with (17). Thus, we have made all the assumptions which lead to (17) and, in addition, κ<1. We can calculate

(34) $$N_{i}=\gamma N_{0}\sum_{j=i}^{\infty }\kappa^{i}=\gamma N_{0}\left(\sum_{j=0}^{\infty }\kappa^{i}-\sum_{j=0}^{i-1}\kappa^{i}\right)=\gamma N_{0}\frac{\kappa^{i-1}}{1-\kappa }$$

Let us set $\epsilon =10^{\delta \beta }$, and we remember that $\kappa =\kappa^{*}\epsilon^{-s}$. Then from (34), we have

$$N\left(M\geq M_{i}\right)=\gamma N_{0}\frac{{\kappa^{*}}^{i-1}\epsilon^{-(i-1)s}}{1-\kappa^{*}\epsilon^{-s}}$$

Next, we assume that $\epsilon =10^{\delta \beta }$. Then from the last relationship, we obtain

$$N\left(M\geq M_{i}\right)=\gamma N_{0}\frac{{\kappa^{*}}^{i-1}10^{-\delta \beta (i-1)s}}{1-\kappa^{*}10^{-\delta \beta s}}$$

Let now $M_{i}=\delta i$. Then,

$$N\left(M\geq M_{i}\right)=\gamma N_{0}\frac{{\kappa^{*}}^{i-1}10^{-M_{i}\beta s}}{10^{-\delta \beta s}\left[1-\kappa^{*}10^{-\delta \beta s}\right]}$$

Furthermore, we set b=βs. In addition, we set $10^{a}=\gamma N_{0}\frac{{\kappa^{*}}^{i-1}}{10^{-\delta \beta s}\left[1-\kappa^{*}10^{-\delta \beta s}\right]}$ Then

(35) $$N\left(M\geq M_{i}\right)=10^{a-bM_{i}}.$$

## 6. The Omori Law

In order to obtain the Omori law, we start from the system (7). We consider the case where $\sigma_{i}\left(t\right)=0$. This means that we have a large earthquake and that no energy moves to the area under consideration. What happens are aftershocks ($\gamma_{i}\left(t\right)\neq 0$) and changes in energy of the sources of seismic activity. ($f_{i}\left(t\right)\neq 0$). The model system becomes

(36) $$\begin{array}{ccc} \frac{dN_{0}}{dt} & = & -\left[f_{0}\left(t\right)+\gamma_{0}\left(t\right)\right]N_{0}\left(t\right).\\ \frac{dN_{i}}{dt} & = & f_{i-1}\left(t\right)N_{i-1}\left(t\right)-\left[f_{i}\left(t\right)+\gamma_{i}\left(t\right)\right]N_{i}\left(t\right),i=1,\ldots . \end{array}$$

Let $N\left(t\right)=\sum_{i=0}^{\infty }N_{(}t)$ be the total number of sources of seismic activity. We sum the equations of the model system. The result is

(37) $$\frac{dN}{dt}=-\sum_{j=0}^{\infty }\gamma_{i}\left(t\right)N_{i}\left(t\right).$$

The key assumption is that $\gamma_{i}\left(t\right)=\gamma \left(t\right)$. Then, the last relationship becomes

(38) $$\frac{dN}{dt}=-\gamma \left(t\right)N\left(t\right).$$

The solution of this equation is

(39) N(t)=Dexp[−∫dtγ(t)],

where *D* is a constant of integration.

Let us first consider the case γ(t)=γ. Then

N(t)=Dexp[−γt].

The number of aftershocks at the moment *t* is

(40) $$N^{*}\left(t\right)=\sum_{j=0}^{\infty }N_{i}^{*}=\sum_{j=0}^{\infty }\gamma N_{i}\left(t\right)=\gamma N\left(t\right)=\gamma D\exp\left[-\gamma t\right].$$

Thus, for the case of constant γ, we have the exponential decay of aftershocks in the considered time. Note that for small *t*,

$$N^{*}\left(t\right)\approx \frac{\gamma D}{1+\gamma t},$$

which is the original form of the Omori law.

In order to obtain the Omori law, we have to assume that γ(t) has appropriate dependence on *t*. Then, we have to start from (39). In this case, the number of aftershocks at the time *t* is

$$N^{*}\left(t\right)=\sum_{j=0}^{\infty }N_{i}^{*}=\sum_{j=0}^{\infty }\gamma \left(t\right)N_{i}\left(t\right)=\gamma \left(t\right)N\left(t\right)=\gamma \left(t\right)D\exp\left[-\int dt\gamma \left(t\right)\right].$$

We equate this to the Omori law (5) and obtain an equation for γ(t). Let us assume that γ(t)=(dμ/dt)/μ. This leads to the following equation for μ:

(41) $$\frac{d\mu }{dt}=\frac{k}{D{\left(c+t\right)}^{p}}\mu^{2}.$$

The solution of this equation is

$$\mu =\frac{D\left(p-1\right){\left(c+t\right)}^{p-1}}{k}.$$

From here,

$$\gamma \left(t\right)=\frac{p-1}{c+t}.$$

In order to obtain the Omori law, we have to set the integration constant *D* to D=k/(p−1). Thus,

(42) $$\gamma \left(t\right)=\frac{k}{D(c+t)}.$$

Thus, the Omori law occurs for the specific value of γ(t) given by (42).

## 7. Earthquakes from Different Clusters of Sources of Seismic Activity

Let us consider (17). For the case of two volumes containing clusters of seismic sources—cluster 1 and cluster 2—we obtain

(43) $$\frac{N_{i}^{\left(1,2\right)}}{N_{0}^{1,2}}=\gamma_{i}^{\left(1,2\right)}{\kappa^{i}}^{\left(1,2\right)}$$

Below, we demonstrate the occurrence of the negative binomial distribution for the observation of earthquakes happening in several clusters of seismic sources. In order to achieve this, we return to the specific case of the relationship (17) considered above in the text. This specific case leads us to the relationship (23). For the case of two clusters, we obtain from (23)

(44) $$\frac{N_{i}^{\left(1,2\right)}}{N_{0}^{\left(1,2\right)}}=q^{i}\left(1-q\right)$$

where *q* is the same for the two regions and *i* runs from 1 to n−1 for region 1 and from 1 to m−1 for region 2. We treat the number of earthquakes in the two regions as independent random variables. Under this assumption (that T=X+Y is a sum of two independent random variables), we obtain for the distribution function $f_{T}=f_{X+Y}\left(t\right)$, t<min(n,m).

fT(t)=∑xfX(x)fY(t−x)=∑x=0tqx(1−q)qt−x(1−q)=t+1tqt(1−q)2.

Then, for this process,

(45) NiN0=i+1iqi(1−q)2.

However,

$$N_{0}=N_{0}^{\left(1\right)}+N_{0}^{\left(2\right)}=\frac{N_{t}^{\left(1\right)}}{1-q^{n}}+\frac{N_{t}^{\left(2\right)}}{1-q^{m}}.$$

Here, $N_{t}^{\left(1\right)}=\sum_{j=0}^{n-1}N_{j}$ for region 1 and $N_{t}^{\left(2\right)}=\sum_{j=0}^{m-1}N_{j}$ for region 2. Then the number of earthquakes generated in the two regions is

(46) Ni=i+1iqi(1−q)2Nt(1)1−qn+Nt(2)1−qm.

For the case of *r* regions, the corresponding result is

(47) Ni=i+r−1r−1qi(1−q)r∑k=1rNt(k)1−qn(k).

Thus, the discussed model supports the assumption [1] and the observations [51,52] that the negative binomial distribution can occur as a statistical distribution of earthquake numbers and can describe very well sets of such numbers.

## 8. Discussion

In this article, we discuss a simple model that leads to several important relationships connected to earthquakes such as the Gutenberg–Richter law and the Omori law. The model uses minimal information about the topology and mechanics of occurrence of earthquakes. Instead, the model is based on the concept of the motion, dissipation, accumulation, and release of energy in a cluster of sources of seismic activity. The sources of seismic activity are distributed in groups according to their internal energy. In the case of an infinite number of groups, we consider a specific form of the model, which is given by the system of Equation (6). This system can be treated numerically. The assumption of a much larger probability of accumulation of internal energy leads us to the system of Equation (7). This system can be solved analytically, and it is the basis for analytical results discussed in this article. In the case of a finite number of groups, we have to add Equation (10). This model can describe various kinds of complicated dynamics. The Gutenberg–Richer law and the Omori law are obtained for specific cases of the model. In order to obtain the Gutenberg–Richter law, we have to assume a specific case where one has a stationary state and time-independent parameters of the model. In addition, we reduced the number of parameters which regulate the motion of energy: we have only three such parameters: γ, σ, and *f*. We note that the relationships are obtained for a single volume (single cluster of sources of seismic activity). In addition, we note that the model can also lead to relationships that are different from the Gutenberg–Richter relationship. This can happen if the values of some of the parameters $\gamma_{i}$, $\sigma_{i}$, and $f_{i}$ differ from the values γ, σ, and *f* or/and the case is not stationary. Thus, the model can explain a part of the differences between the relationship connected to the real situation and the Gutenberg–Richter relationship, which is connected to very specific requirements about the model parameters and about the motion of the energy in the (single) cluster of sources of seismic activity.

The Omori law is obtained for the specific case of the absence of an exchange of energy between the environment and the system of seismic sources after a large earthquake. In addition, a specific value of the parameter γ(t), which regulates the release of seismic energy, must be presented, and this parameter has to be the same for the sources of seismic activity with different energies. It is clear that if these requirements are not met, the relationship for the time dependence of the number of aftershocks can differ from the Omori law, and this law can be only an approximation of the relationship corresponding to the real situation.

Finally, we discuss the distribution of earthquakes from several volumes (several clusters of sources of seismic activity). We show that the distribution of earthquakes in this case contains a specific case distribution which is very close to the negative binomial distribution. The obtained distribution has two parameters. The first parameter *r* is characteristic of the number of volumes (clusters of sources of seismic activity), and the second parameter *q* can be connected to the parameter *b* of the Gutenberg–Richter law. The occurrence of a distribution that is close to the negative binomial distribution leads to a natural explanation of the roll-off effect and the possibility for the occurrence of characteristic earthquakes (large-magnitude earthquakes which occur more frequently as predicted by the Gutenberg–Richter law). Thus, the model supports the hypothesis that the negative binomial distribution occurs because of the consideration of earthquakes from more than one cluster of sources of seismic activity.

Further information about the application of the theory to real data can be seen in Appendix A.

## 9. Conclusions

In this article, we present a simple mathematical theory which leads to several well-known relationships such as the Gutenberg–Richer law, Omori law, etc. The theory is based on the concept of energy. This allows us to obtain the corresponding relationships without the use of considerations connected to the mechanics of seismic events. We do not use details of seismic sources or equations of motion, and we do not consider the different kinds of waves traveling at the surface and in the interior of the Earth. We do not use details about kinematics or about the dynamics of the seismic sources. However, we have shown that a simple model, linear with respect to N(t) and with time-dependent coefficients in general, is capable of leading to important laws and relationships in seismology. We note that research based on energy considerations has also been conducted by other authors [53].

We think that our approach will point to new research questions. One of these questions is about the connections between the presented theory and the characteristics of the mechanical vibrations of the Earth caused naturally by earthquakes. Other questions are connected to the relationship between the presented theory and the aspects of the mechanics of seismic events, mentioned above. Research results on some of these questions will be presented elsewhere.

## Data Availability

He original contributions presented in the study are included in the article, further inquiries can be directed to the corresponding author.

## Appendix A. Notes About the Application of the Model (6) to Real Data

The model (6) and its truncated versions (6), (9), can fit very well into any real data set because of the flexibility of the model parameters. To show this, we present below a Lemma and a Theorem that prove the last statement for a single cluster of sources of seismic activity (the Lemma) and any set of clusters of sources of seismic activity (the Theorem).

**Lemma** **A1.**
*Let us have a cluster of sources of seismic activity, and let us measure the changes in the internal energy of the sources over time. Let the internal energy of the sources in the group i be between $E_{i-1}$ and $E_{i}>E_{i-1}$, i=1,2,…. Let us record the following: the rise of new sources of seismic activity, changes in the internal energy of existing sources, and the release of internal energy by means of earthquakes. Then, the model (6) describes the time evolution of the number of sources of seismic activity in each group of sources of seismic activity.*

**Proof.** Let us consider the groups of sources of seismic activity that occur from sorting the sources in a cluster of sources of seismic activity according to their internal energy, as described in the text of the Lemma. Then, we consider the following:

1. One observes the birth of some number of new sources of seismic activity in each group. This change is described in the model by the term $\sigma_{i}\left(t\right)N_{i}\left(t\right)$. Let the number of the newborn sources be $N_{i}^{*}\left(t\right)$. Then we can set $\sigma_{i}\left(t\right)=N_{i}^{*}\left(t\right)/N_{i}\left(t\right)$. Thus, the model (6) captures correctly the number of new sources of seismic activity arising in each group *i* at any moment *t*.
2. The internal energy of the sources of seismic activity from group *i* can increase and decrease in time and some of these sources can leave group *i*. The model gives the number of sources of seismic activity that increase their internal energy and leave group *i* as $f_{i}\left(t\right)N_{i}\left(t\right)$ and the number of sources of seismic activity that decrease their energy and leave group *i* as $g_{i}\left(t\right)N_{i}\left(t\right)$. Let the number of sources of seismic activity that increase their internal energy and leave group *i* be $I_{i}\left(t\right)$. Let the number of sources of seismic activity that decrease their internal energy and leave group *i* be $D_{i}\left(t\right)$. We can set $f_{i}\left(t\right)=I_{i}\left(t\right)/N_{i}\left(t\right)$ and $g_{i}\left(t\right)=D_{i}\left(t\right)/N_{i}\left(t\right)$. Thus, the model (6) captures correctly any changes in the number of the existing sources of seismic activity in group *i* which are a consequence of an increase or decrease in the internal energy of the sources, which leads to the transfer of sources of seismic activity to other groups.
3. Finally, the number of sources of seismic activity in group *i* can decrease because of the release of energy by the sources in the form of earthquakes. The model describes this change as $\gamma_{i}\left(t\right)N_{i}\left(t\right)$. Let the number of earthquakes from the *i*-th group be $Q_{i}\left(t\right)$. Then we can set $\gamma_{i}\left(t\right)=Q_{i}\left(t\right)/N_{i}\left(t\right)$. Thus, the model (6) captures correctly any changes in the number of the existing sources of seismic activity in group *i* which are a consequence of the release of internal energy in the form of earthquakes.

Thus, we have shown that the model (6) can capture correctly the birth, deaths, and changes in the number of sources of seismic activity with a specified internal energy in a cluster of sources of seismic activity using the appropriate setting of the functions $\sigma_{i}\left(t\right)$, $f_{i}\left(t\right)$, $g_{i}\left(t\right)$, and $\gamma_{i}\left(t\right)$, which are parametric functions of the model. □

**Theorem** **A1.**
*Let us have a set of clusters of sources of seismic activity. Let us measure the changes of the internal energy of these sources over time. Let us group the sources of seismic activity from any cluster k (k=1,2,…) in groups, and let the internal energy of the sources in group i be between $E_{k_{i-1}}$ and $E_{k_{i}}>E_{k_{i-1}}$, i=1,2,…. Let us record the following: the rise of new sources of seismic activity, changes in the internal energy of the existing sources, and the release of internal energy through earthquakes in any of the clusters. Then, the model (6) describes the time evolution of the number of sources of seismic activity in each group of such sources in the entire set of clusters of sources of seismic activity.*

**Proof.** According to the Lemma above, the model (6) describes the time evolution of the number of sources of seismic activity in each group of such sources in any of the clusters of sources of seismic activity. This holds for any cluster from the sets of clusters. Thus, the model (6) describes the time evolution of the number of sources of seismic activity in each group of energy sources in any cluster of the entire set of clusters of sources of seismic activity. □

We note that a similar Lemma and Theorem can be formulated also for the truncated version of the model (6), which includes Equations (6) and (9). Thus, the model discussed in the main text is very flexible for describing the corresponding class of experimental sets of data connected with seismic activity.
